# Phenotypic characterization and genomic analysis of a Salmonella phage L223 for biocontrol of *Salmonella* spp. in poultry

**DOI:** 10.1038/s41598-024-64999-1

**Published:** 2024-07-03

**Authors:** Md Abu Sayem Khan, Zahidul Islam, Chayan Barua, Md. Murshed Hasan Sarkar, Md. Firoz Ahmed, Sabita Rezwana Rahman

**Affiliations:** 1 Department of Microbiology, University of Dhaka, Dhaka, Bangladesh; 2 Genomics Research Laboratory, Bangladesh Council of Scientific and Industrial Research, BCSIR, Dhaka, 1205 Bangladesh; 3 Department of Microbiology, Jahangirnagar University, Savar, Dhaka, Bangladesh

**Keywords:** *Salmonella*, Lytic bacteriophage, Biocontrol, Poultry, Whole-genome sequence, Bacteriophages, Antimicrobial resistance

## Abstract

The escalating incidence of foodborne salmonellosis poses a significant global threat to food safety and public health. As antibiotic resistance in *Salmonella* continues to rise, there is growing interest in bacteriophages as potential alternatives. In this study, we isolated, characterized, and evaluated the biocontrol efficacy of lytic phage L223 in chicken meat. Phage L223 demonstrated robust stability across a broad range of temperatures (20–70 °C) and pH levels (2–11) and exhibited a restricted host range targeting *Salmonella* spp., notably *Salmonella* Typhimurium and *Salmonella* Enteritidis. Characterization of L223 revealed a short latent period of 30 min and a substantial burst size of 515 PFU/cell. Genomic analysis classified L223 within the *Caudoviricetes* class, *Guernseyvirinae* subfamily and *Jerseyvirus* genus, with a dsDNA genome size of 44,321 bp and 47.9% GC content, featuring 72 coding sequences devoid of antimicrobial resistance, virulence factors, toxins, and tRNA genes. Application of L223 significantly (*p* < 0.005) reduced *Salmonella* Typhimurium ATCC 14,028 counts by 1.24, 2.17, and 1.55 log CFU/piece after 2, 4, and 6 h of incubation, respectively, in experimentally contaminated chicken breast samples. These findings highlight the potential of Salmonella phage L223 as a promising biocontrol agent for mitigating *Salmonella* contamination in food products, emphasizing its relevance for enhancing food safety protocols.

## Introduction

*Salmonella,* a Gram-negative, foodborne zoonotic pathogen, poses a substantial threat to food production and public health globally^1^. Consumption of *Salmonella*-contaminated foods leads to salmonellosis, an acute infection characterized by symptoms such as fever, nausea, vomiting, and abdominal pain^2^. Non-typhoidal salmonellosis (NTS) accounts for approximately 90 million cases of food poisoning and 155,000 deaths worldwide annually, representing a serious threat to human health^3,4^. In the European Union alone, *Salmonella* is the most frequently isolated pathogen in foodborne outbreaks, contributing to nearly 91,000 cases of salmonellosis annually^5^. Contaminated food items play a major role in around 50% of NTS infections, with fresh-cut produce, raw or undercooked meat, poultry, and eggs being commonly implicated^6^. The prevalence of *Salmonella* in food varies based on factors such as country, region, food production methods, cultural practices, and geographic location^7^. Furthermore, *Salmonella* infections can vary in severity depending on the health status of the human host and the serotype involved^8^. Among over 2600 known serotypes of *Salmonella*, Enteritidis and Typhimurium are the most common food-related serotypes, accounting for half of all salmonellosis cases^9,10^.

Although antibiotics are frequently used to control *Salmonella* infections in humans and veterinary settings, their excessive use has led to an increase in the emergence and transmission of multidrug-resistant (MDR) bacteria over the past decades^11^. In terms of temporal distribution, the prevalence of antimicrobial-resistant (AMR) *Salmonella* increased from 53 to 77% in South Asia between January 2010 to June 2021^12^. The increase in the occurrence of ESBL-producing non-typhoidal *Salmonella* in animal and poultry products has become of great concern due to resistance to cephalosporins that are used to treat severe salmonellosis in humans^13^. In addition, the emergence and dissemination of mobilizable and plasmid-borne *mcr* genes mediated colistin resistance in *Salmonella enterica* in humans and livestock across different countries threatens the efficacy of colistin, a last-resort antibiotic of the polymyxin family that is indicated for treating invasive infections by multidrug-resistant *Enterobacteriaceae* in humans^14^. According to epidemiological studies, MDR strains of *Salmonella* are more virulent than sensitive strains because they cause infections to become more severe^15^. The occurrence of MDR *Salmonella* not only causes economic loss but also complicates the control of foodborne outbreaks, potentially increasing the number of hospitalizations and mortality rates. Therefore, the need for new antimicrobial agents is of utmost importance to combat *Salmonella* infections in humans and livestock.

Bacteriophages, the bacteria-killing viruses, have gained renewed attention as an antibacterial for controlling pathogens in food, health and agriculture due to their bactericidal capability, availability in the environment, ease of isolation and cost-effective production characteristics^16^. The unique properties of phages, including their capacity to selectively kill targeted pathogens, non-toxicity to humans, self-replication and self-limiting, make them suitable for use in food safety applications^17^. Moreover, phage treatment of bacterial contamination has some potential advantages over conventional use of antimicrobials. Phages do not harm beneficial microbes in foods and human and animals’ intestinal tracts. Next, it is unlikely that the addition of phages causes negative impacts on food characteristics^18^. A growing body of literature on the isolation, characterization and application of *Salmonella* phages reported some notable findings in favor of their effectiveness in reducing *Salmonella* load on a wide variety of foods including eggs, chicken meat, sausage, milk, seafood, lettuce, tomatoes and others. Islam et al. 2019, reported a significant 3log CFU reduction in the viable count of *Salmonella* in milk and chicken breast after phage treatment at 4 °C and 25 °C. They also observed the anti-biofilm effect of *Salmonella* phage on microtiter plates and stainless steel^19^. Another study found that application of *Salmonella* phage LPSE1 at MOI = 1 had reduced the number of recoverable *Salmonella* by 1.44, 0.52, and 2.02 log CFU on milk, sausage and lettuce, respectively at MOI = 1^20^. A recent study by Park et al., observed that the treatment of chicken milk and meat with phage MSP1 reduced the number of *Salmonella* below the detection limit^21^. Such promising findings also led to the development of phage-based products. The US Food and Drug Administration (FDA) and the US Department of Agriculture (USDA) have officially approved several commercial phage products for use in food to combat foodborne pathogens such as *Salmonella* spp., *E coli* and *Listeria monocytogenes*^22^. Intralytix Inc, USA developed a cocktail of six phages, SalmoFresh that got FDA approval for direct use in poultry, fish and shellfish, fresh and processed fruits, and vegetables. Salmonelex is another commercial phage preparation that was approved in 2013 as an authorized processing aid for the manufacture of meat and poultry products. In addition, Listex P100 has been granted GRAS (Generally Recognized As Safe) status by the US FDA and endorsed by the Food Safety and Inspection Service for its antimicrobial use in ready-to-eat meat and poultry items^23^. However, the use of phages often has geographical limitations due to the narrow lytic range and distribution of serotypes and therefore, the local scale isolation and application of new phages is necessary^24^.

Salmonellosis remains a major concern in the growth of the poultry industry in Bangladesh with a prevalence of between 28% and 53.3%^25^. In recent years, numerous studies reported the higher occurrence of MDR *Salmonella* with plasmid-mediated resistance genes in poultry processing environments and related products^26–28^. Such reduced susceptibility to different commonly used antibiotics in poultry due to their indiscriminate use develops treatment failure^29^. In this regard, phages can be a well-suited antibacterial technology for food decontamination, veterinary applications and human treatment in lower- and middle-income countries (LMIC), as Bangladesh^30^. While previous studies in Bangladesh have focused on isolating and characterizing *Salmonella* phages at a physicochemical level, a comprehensive assessment of their genomic characteristics and suitability for biocontrol applications remains lacking. Recognizing the potential of bacteriophages against antimicrobial-resistant (AMR) *Salmonella* strains, we isolated a *Salmonella* phage (L223) from poultry sewage and conducted detailed analyses of its physicochemical stability, replication kinetics, and genomic properties. Furthermore, we assessed the biocontrol efficacy of the isolated phage against *Salmonella* Typhimurium in chicken meat, aiming to provide valuable insights into its practical application in food safety and public health initiatives.

## Results

### Phage isolation and characterization

**Figure 1:**
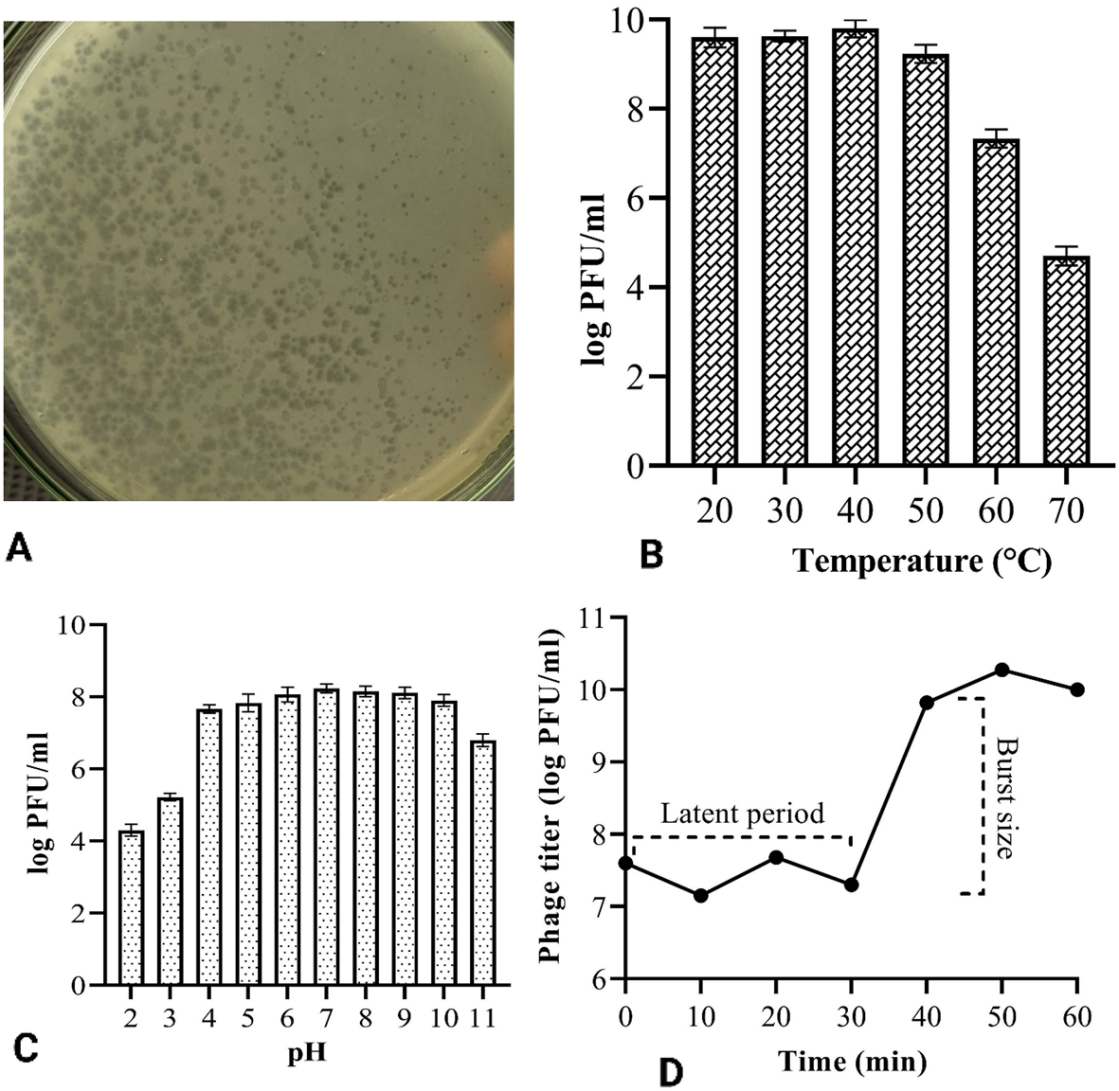
Different characteristics of Salmonella phage L223. (**A**) Lytic plaque on soft agar; (**B**) Temperature stability of L223; (**C**) Phage stability at different pH and (**D**) One step growth curve.

*Salmonella* phage L223 was isolated from a poultry market sewage sample that produced small (approximately 2 mm), round and lytic plaque on the lawn of *Salmonella* Typhimurium ATCC14028 (Fig. 1A). Following isolation, propagation and purification, a stock titer was prepared maintaining a concentration of 10^10^ PFU/ml. The latent period and burst size of L223 were estimated to be 30 min and 515 PFU/cell, respectively (Fig. 1D). It showed stability to a wide range of temperature and pH. Though the optimum temperature was 40 °C, no noticeable variation in phage stability was observed between 20 °C and 40 °C (Fig. 1B). After that, phage counts tended to decrease and no activity was found at 80 °C. Meanwhile, subjecting phage at pH between 2 and 11 exhibited that phage can tolerate this wide spectrum of acid–base changes. The average phage count at pH between 4 and 10 was 7.9 ± 0.25 log PFU/ml (Fig. 1C).

Isolated phage also infected 3 *Salmonella* Typhimurium, 2 *Salmonella* Enteritidis and 2 undetected *Salmonella* serotypes that were previously isolated from different poultry samples. The EOP of phage L223 in *Salmonella* Typhimurium and *Salmonella* Enteritidis ranged from 0.52 to 0.80. However, no lytic activity against bacteria from other species including, *E. coli* ATCC 25922, *Pseudomonas aeruginosa, Vibrio cholerae, Shigella flexneri, Acinetobacter baumanii,* and *Staphylococcus aureus* suggested a narrow host range of phage L223 (Table 1).

**Table 1:** Host range of Salmonella phage L223.

Bacteria	Strain ID	Source (location)	Phage lysis	EOP
*Salmonella* Typhimurium	S5	Poultry excreta	+	0.80
*Salmonella* Typhimurium	K7	Eggshell	+	0.72
*Salmonella* Typhimurium	K10	Chicken carcass	+	0.60
*Salmonella* Enteritidis	E2	Eggshell	+	0.75
*Salmonella* Enteritidis	K8	Eggshell	+	0.52
*Salmonella* spp.	S9	Chicken carcass	+	0.20
*Salmonella* spp.	K2	Chicken carcass	+	0.04
*E. coli*	ATCC 25922	ATCC	−	0
*E. coli*	S2C	Clinical		0
*E. coli*	S11C	Clinical		0
*Pseudomonas aeruginosa*	W5D	Wastewater	−	0
*Pseudomonas aeruginosa*	S3C	Clinical	−	0
*Pseudomonas aeruginosa*	S5C	Clinical	−	0
*Vibrio cholerae*	SF1SM	Street food	−	0
*Vibrio cholerae*	SF3SH	Street food	−	0
*Vibrio cholerae*	DS1	Pond water	−	0
*Shigella flexneri*	DS5	Street food	−	0
*Shigella flexneri*	W9SS	Wastewater	−	0
*Shigella flexneri*	W2DMC	Wastewater	−	0
*Acinetobacter baumanii*	S1C	Clinical	−	0
*Acinetobacter baumanii*	S10C	Clinical	−	0
*Acinetobacter baumanii*	S52C	Clinical	−	0
*Staphylococcus aureus*	SA23	Street food	−	0
*Staphylococcus aureus*	SA21C	Clinical	−	0
*Staphylococcus aureus*	SA50C	Clinical	−	0

+ indicated lysis, and − indicated no lysis.

**Figure 2:**
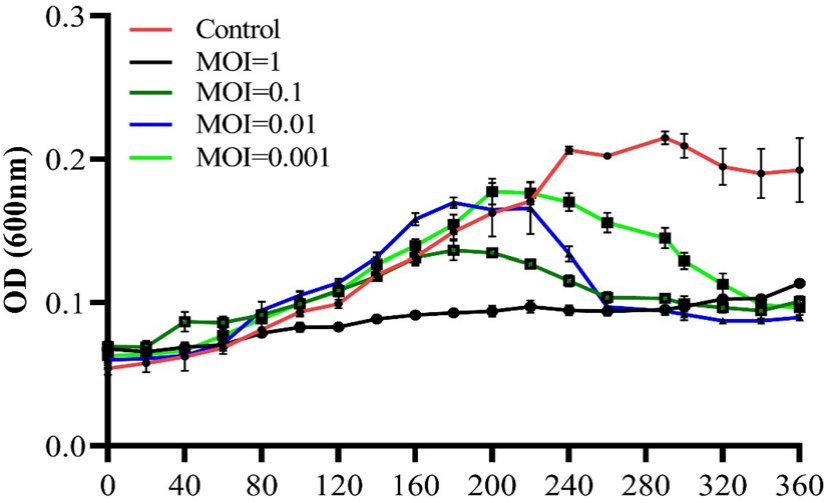
Bacteriolytic kinetics of Salmonella phage L223 against *Salmonella* Typhimurium.

In vitro bacteriolytic activity of phage L223 was investigated through time-kill curve analysis at different MOIs (1, 0.1, 0.01, and 0.001) up to 360 min (6 h). Compared to phage-free control, bacteriolytic activity was observed at all MOIs based on the measurement of optical density. The optical density at MOI = 1 remained consistent throughout the experimental time, whereas bacterial growth initially increased and tended to decrease after 4 h as evidenced by a reduction in optical density at MOI = 0.1, 0.01 and 0.001 (Fig. 2).

### Phage genome analysis

**Figure 3:**
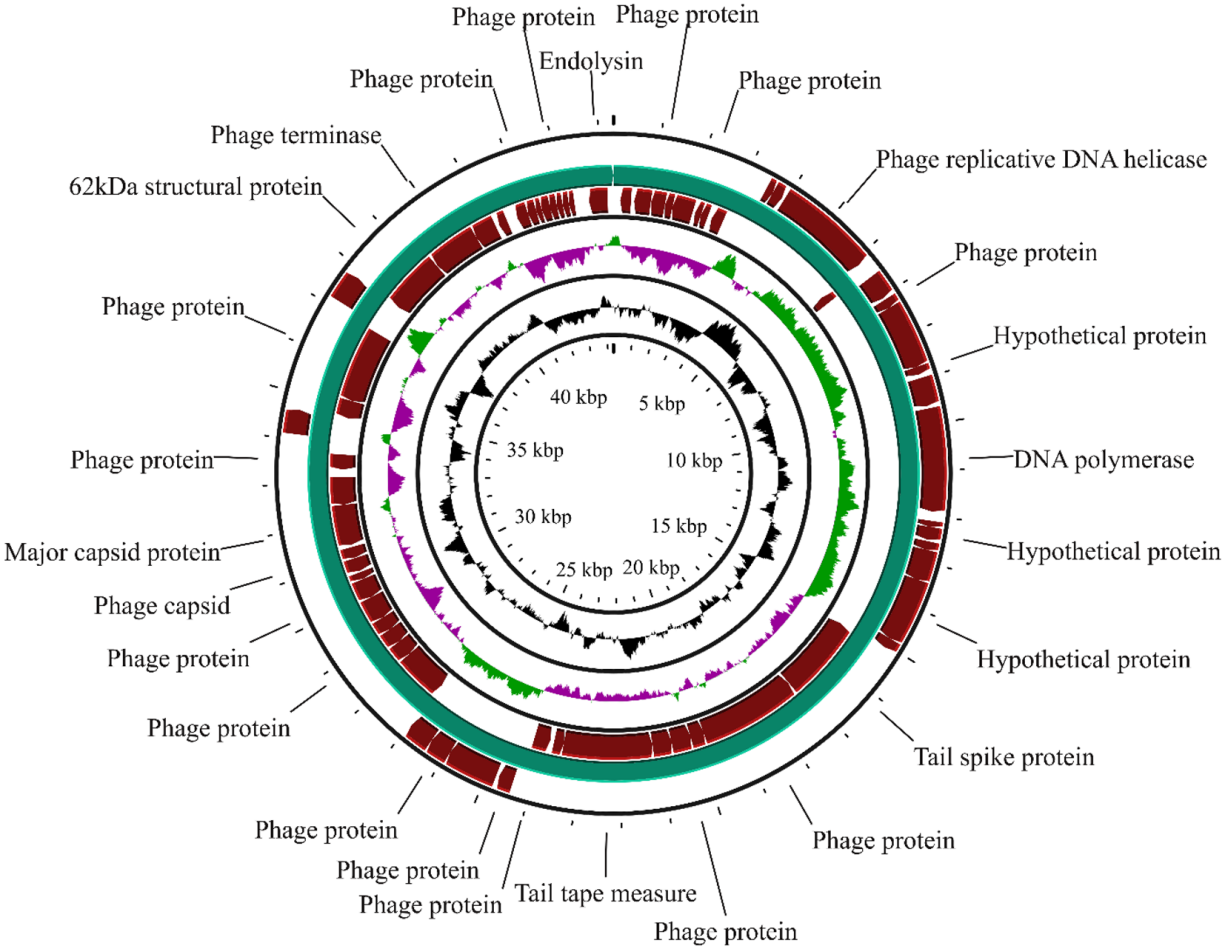
Circular genome map of Salmonella phage L223.

The length of the phage genome was 44321 bp of circular dsDNA with 47.9% GC content. According to CheckV, the completeness was 100% (GenBank Accession no.: PP034127). RAST-based genome annotation predicted 72 coding sequences (CDS). Based on the predicted roles of these CDSs, the proteins can be distributed into structural proteins (capsid protein, tail spike protein), replication and regulatory proteins (DNA polymerase, DNA helicase), packaging-associated proteins (terminase) and host cell lysis proteins (holin, endolysin) (Fig. 3). The phage genome had no genes for antimicrobial resistance, virulence and tRNA. The lifestyle of the phage, as predicted by BACPHLIP, was lytic (virulent) with a score of 0.9875.

### Taxonomic assignment, phylogeny and comparative genomic analysis

**Figure 4:**
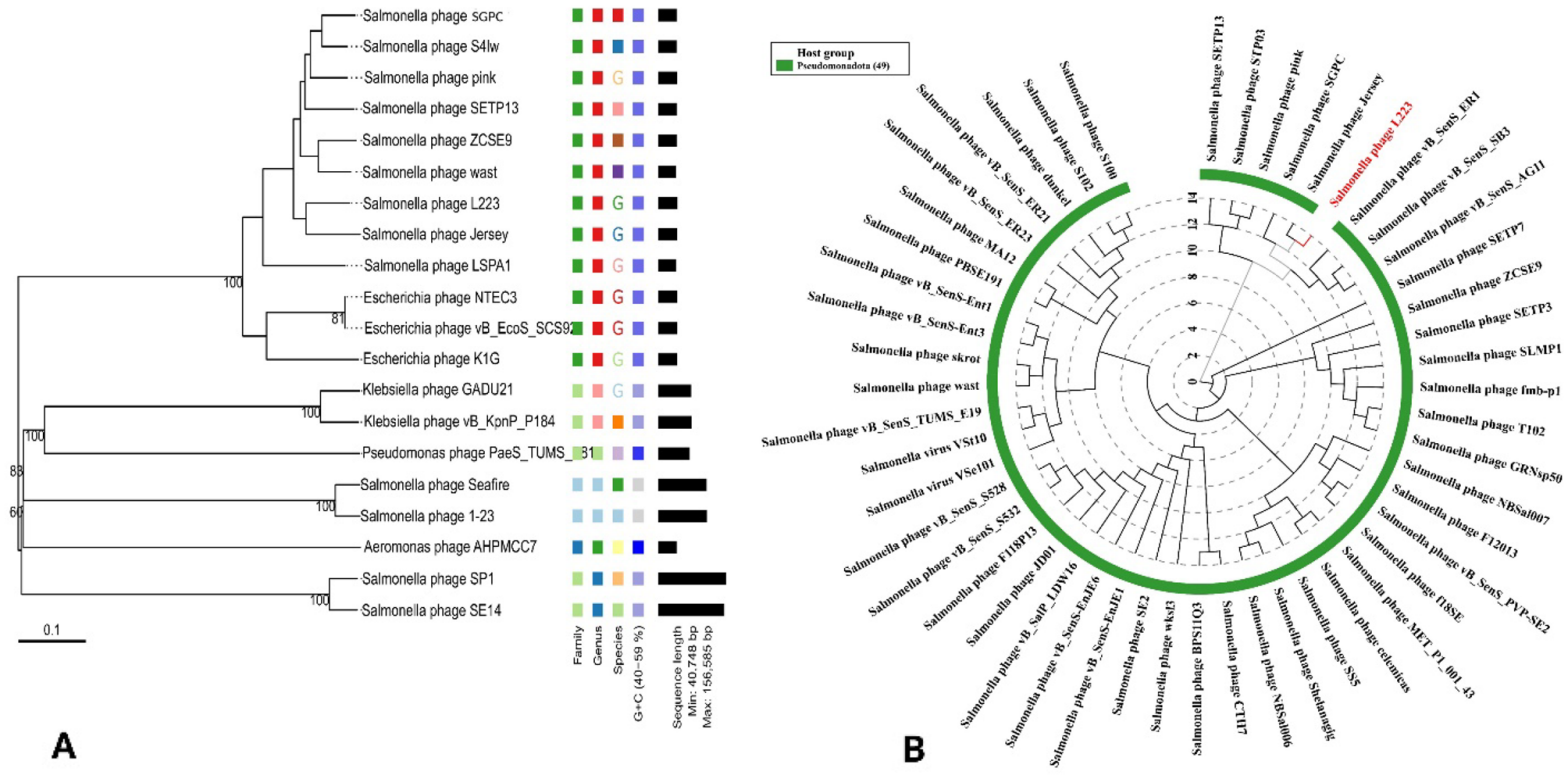
Phylogenetic analysis of Salmonella phage L223 based on (**A**) genome-BLAST Distance Phylogeny (GBDP) and (**B**) proteomic tree based on genome wide similarities.

**Figure 5:**
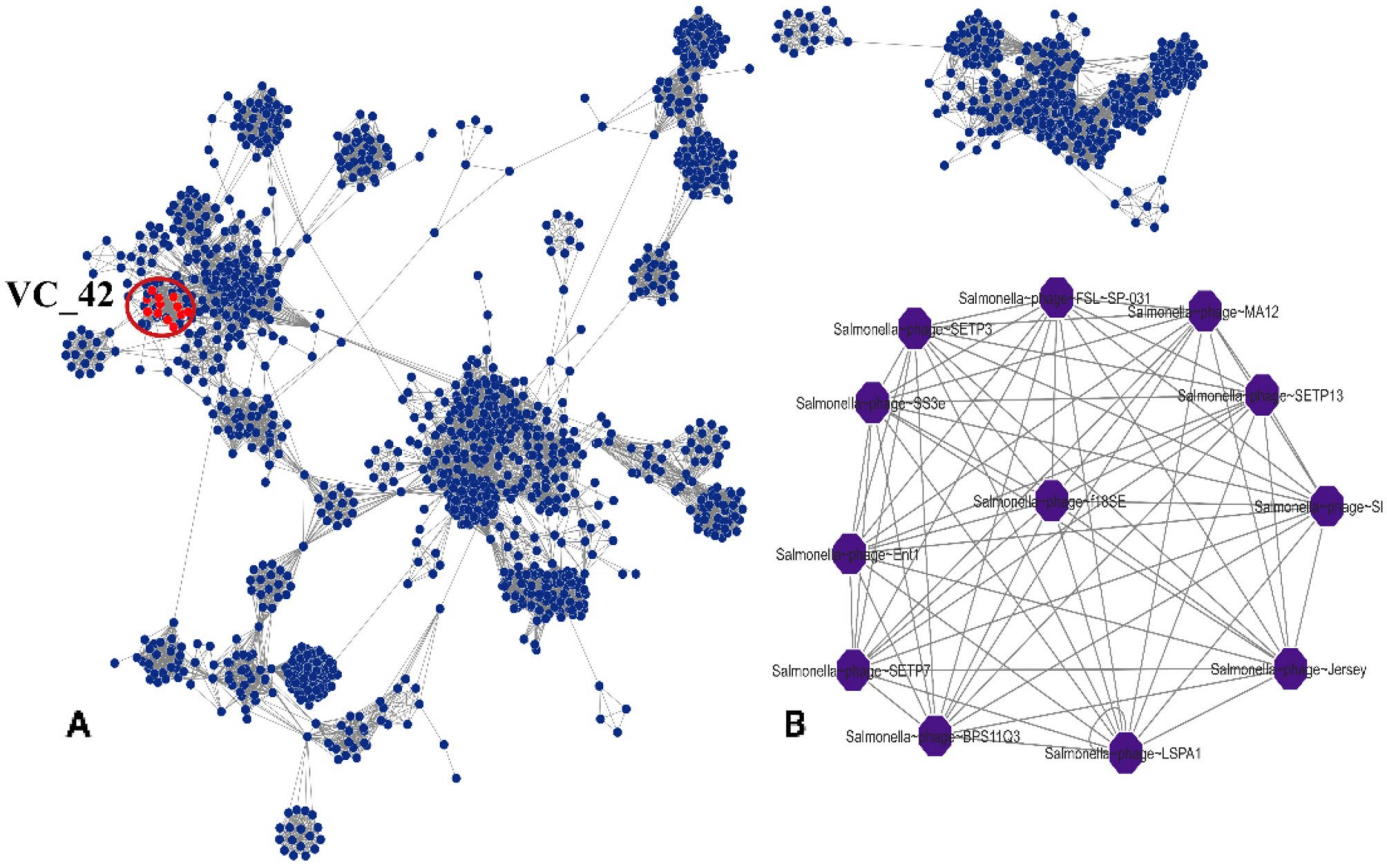
Phage genome network analysis using vConTACT2. (**A**) Salmonella phage L223 occurs in viral Cluster VC_42 (red colored), (**B**) A closer look on VC_42 cluster.

Genome-wide analysis for taxonomic annotation was enabled by VICTOR and vConTACT2. VICTOR-based whole genome phylogeny predicted that *Salmonella* phage L223 shared close relation with phages belonging to the Jerseyvirus genus including phage SGPC, S4Iw, pink, SETP13, ZCSE9, wast and jersey (Fig. 4). A proteome clustering and network analysis using vConTACT2 designated the L223 phage to viral cluster VC_42 (Fig. 5). Phages of this cluster belong to the order: *Caudoviricetes* class, *Guernseyvirinae* subfamily and *Jerseyvirus* genus. The results of VICTOR and vConTACT2 were further reaffirmed by ViPTree and PhageClouds network analysis. The *Salmonella* phage L223 was predicted to be closely linked to *Salmonella* phage Jersey, according to a maximum likelihood tree constructed utilizing viral genomes in ViPTree against dsDNA of all genomes. Moreover, based on the computational analysis of the distance relationship between L223 and other phage genomes deposited in NCBI GenBank, L223 was found to be linked to 116 phage genomes at a distance threshold of ≤ 0.2**.** Similar to other tools**,** Phage Cloud also assigned our isolated phage in the *Jerseyvirus* genus.

**Figure 6:**
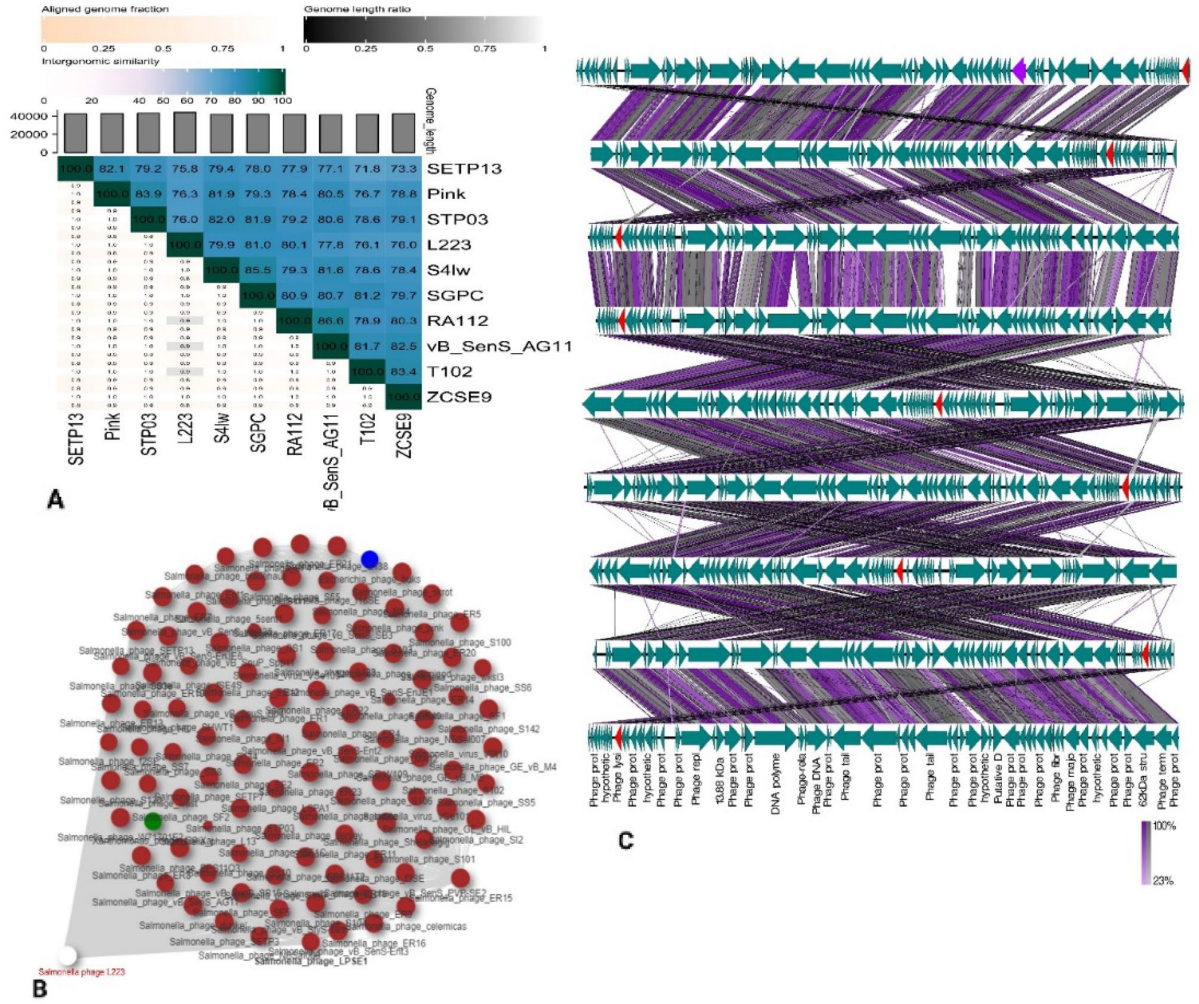
Comparative genomic analysis of Salmonella phage L223 and closely related phage genomes (**A**) VIRIDIC heatmap, (**B**) Whole genome comparison, and (**C**) PhageCloud analysis.

The linear comparison of phage genomes using Easyfig and intergenomic comparisons with VIRIDIC showed similarity between L223 and top hit phages from blastn search. The intergenomic similarity between L223 and closely related homologs ranged from 75.8 to 81%, suggesting that L223 shared the same genus (> 70%) with compared phages but belonged to different species (< 95%). This finding reaffirmed the result of VICTOR. Even, none of the phages used in comparative genomic analysis had 95% similarity with other phages, indicating they all belong to different species (Fig. 6).

### *Pan*-genome analysis

**Figure 7:**
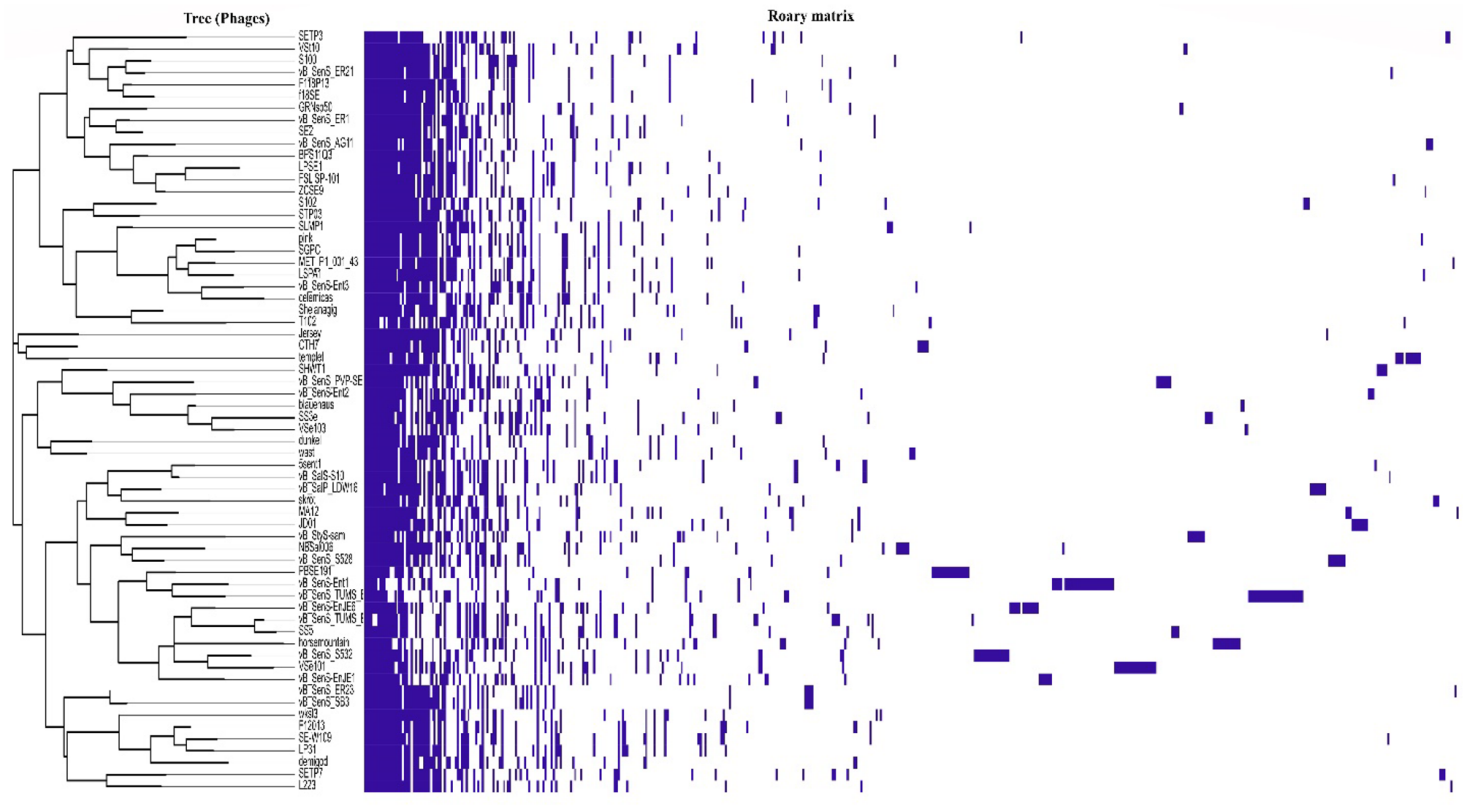
Pan-genome matrix of 65 Salmonella phages.

**Figure 8:**
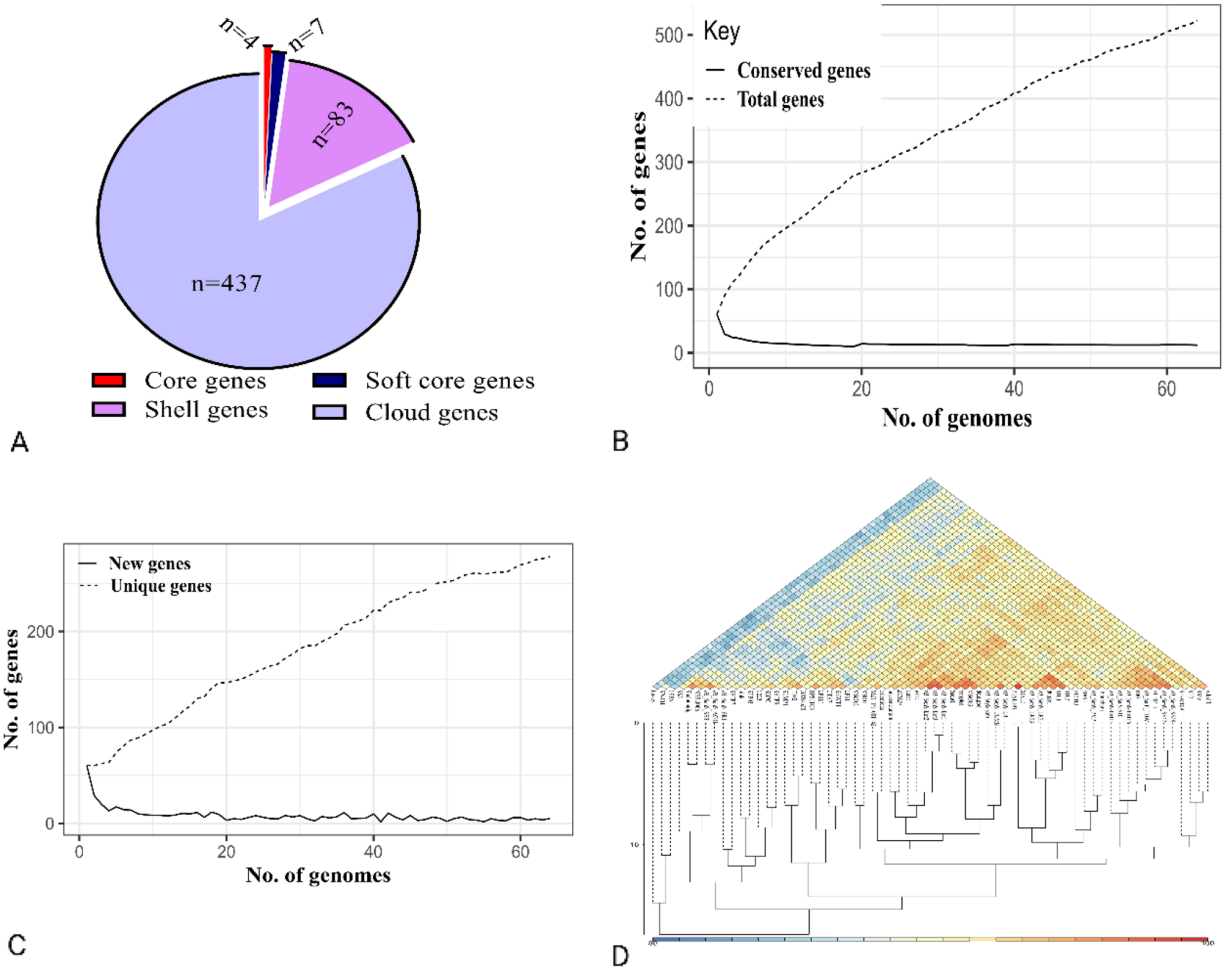
Pan-genome analysis of Salmonella phages: (**A**) Distributions of genes in pan-genome, (**B**) Changes in the number of conserved and total genes, (**C**) Changes in the number of new and unique genes, and (**D**) Average nucleotide identity between phages.

Pan-genome analysis of Salmonella phages indicated the presence of 4 core genes (99% ≤ strains ≤ 100%), 7 softcore genes (95% ≤ strains < 99%), 83 shell genes (15% ≤ strains < 95%), and 437 (0% ≤ strains < 15%) cloud genes (Fig. 7). Moreover, the openness of the pan-genome was evident from the core-pan rarefaction curve (Fig. 8). With the addition of the *Salmonella* phage genome, the number of pan-genome genes increased gradually. The average nucleotide identity of phages remained between 80 and 100%.

### Effect of phage treatment on *Salmonella* Typhimurium on chicken meat

**Figure 9:**
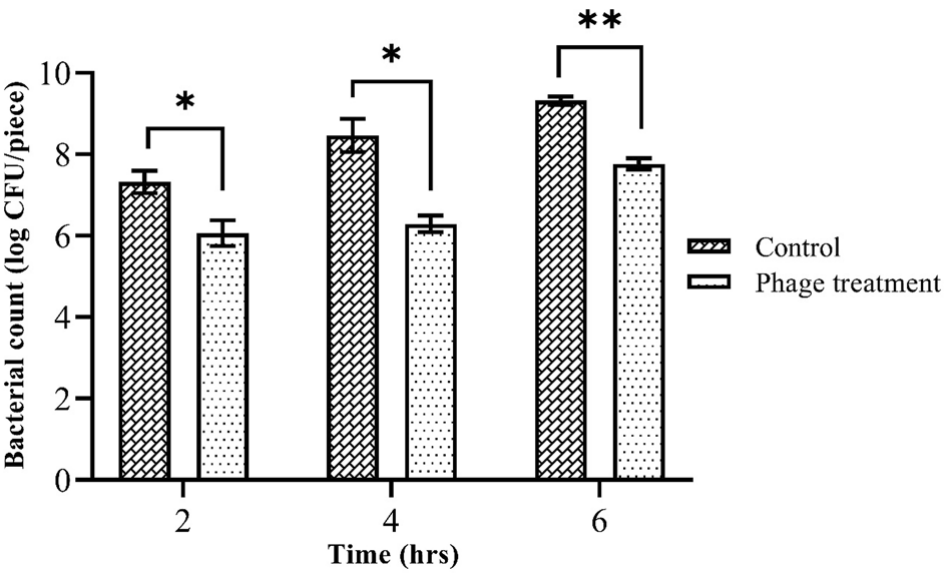
The activity of the phage L223 against *Salmonella* Typhimurium on chicken meat.

The biocontrol activity of isolated phage was examined on *Salmonella* Typhimurium ATCC 14028 contaminated chicken breast samples at 25 °C for 2, 4 and 6 h. We observed that the number of hosts was significantly reduced (*p* < 0.005) upon phage treatment of experimentally contaminated chicken breast compared to phage untreated control during different incubation times (Fig. 9). After 2 h, phage treatment reduced *Salmonella* count by 1.24 log CFU/piece compared to non-treatment. However, a maximum of 2.17 log CFU/piece reduction of *Salmonella* was achieved after 4 h of treatment. Though, the bacterial counts in the phage treated samples at 6 h of incubation were increased, bacterial viabilities were still significantly (*p* < 0.005) lower in the treatment group than those in the phage free control samples.

## Discussion

The worldwide increasing prevalence of foodborne salmonellosis, the treatment failure associated with the emergence of MDR *Salmonella* in veterinary and foods, and the slow progress in the development of new antibiotics have stimulated the efforts to search for alternatives to antibiotics^31^. Lytic phages have now been considered one of the most promising alternatives due to host specificity, ease of application, eco-friendly and cost-effective than antibiotics^32^. A large collection of bacteriophages, the use of obligately lytic phages rather than temperate phages, host range, and screening of phage genomes to validate the lack of toxin genes are some of the characteristics necessary for successful phage therapy in practice^33^. Here, we described the isolation, identification, phenotypic and genotypic characterization, and antibacterial application of a lytic *Salmonella* phage L223 from poultry environmental sewage in Bangladesh.

Temperature resistance and acid–base changes are some of the most essential characteristics of phage utilized in biocontrol applications in foods and the environment^34^. Phage L223 exhibited a broad range of pH (2–11) and thermal stability (≤ 70 °C). The result is consistent with other studies that also reported similar stability in *Salmonella* phage SWJM-01 and SWJM-02, PSDA-2, PS5, ZCSE-9 and others^3,31,35,36^. Such stability at a wide temperature and pH range indicated the suitability of L223 for use in food processing and utilization in foods with different pH values (meat, milk, fruits). The latent period and burst size are two crucial parameters in the assessment of phage fitness and the selection of suitable candidate phages for biocontrol and therapeutic applications^37^. Phage L223 exhibited a short latent period (30 min) and a large burst size of L223 (515 PFU/cell) in one step growth curve. This latent time was similar to phage phiPT1, STGO-35-1, AUFM_Sc3 but shorter than a cocktail of four phages including vB_SnwM_CGG4-1, vB_SnwM_CGG4-2, vB_SnwM_CGG3-1 and vB_SnwM_CGG3-2 (53 to 65 min), P22 (45 min) and st104a (60 min)^38–42^. Moreover, the burst size of L223 was comparable to BPSELC-1 (500 PFU/cell) but larger than vB_SalP_TR2 (211 PFU/cell), OSY-STA (176 pfu/cell), OSY-SHC (256 PFU/cell), SHWT1 (150 PFU/cell) and others^43–45^. This comparison highlights L223's efficiency in phage replication and release compared to other known phages. Bacteriophages with a broad host range are typically preferred in biocontrol applications, even though there is a chance of unfavorable side effects against non-target species. According to the host range analysis, L223 lysed all seven *Salmonella* spp. including *Salmonella* Typhimurium and *Salmonella* Enteritidis but did not produce plaque against bacteria from other genus, suggesting the specificity of this phage. Serovars Typhimurium and Enteritidis were reported to be the predominant serotypes in poultry and poultry products in Bangladesh^26,46^. In addition, mixing different phages, commonly known as phage cocktail, is now the most used method to broaden the host range of phages^31^. The physicochemical stability and multiplication kinetics of L223 suggested that it may have the potential to be used in the formulation of a phage cocktail.

A comprehensive analysis of the phage genome is essential to assess the safety of phages before their use as antibacterial agents. The contribution of phages in the transmission of AMR genes and virulence factors by horizontal gene transfer has been extensively studied. Therefore, phages with AMR and virulence genes are not considered for biological control of pathogens in the food industry. In this regard, whole-genome sequencing can enable the in-depth characterization of phages and thus ensure the security and efficacy of phages for biocontrol applications^47^. The complete genome sequence of L223 consisted of 44321 bp that contained 72 CDS. The genome length of L223 was in alignment with other phages (PSDA-2, ZCSE-9, STGO-35, vB_SenS_TUMS_E4, vB_SenS_SE1) in which the genome size ranged from 40 to 50 kb^31,36,39,48,49^. The predicted CDSs were associated with DNA replication, phage assembly and packaging, structural proteins, host cell lysis and other functional domains. In addition to the observation of lytic plaque in soft agar, the presence of holin and endolysin in the phage genome and the prediction of BACPHLIP confirmed the virulent (lytic) nature of L223. Most importantly, the whole genome analysis also confirmed the absence of antibiotic resistance genes, virulence genes, tRNA and lysogenic genes. Therefore, it can be concluded that L223 does not integrate the gene fragments into the host bacteria's genome during its life cycle and does not cause harm to the host when used to treat *Salmonella* infections. Taxonomic assignment of L223 using VICTOR predicted that it belongs to the *Caudoviricetes* class, *Guernseyvirinae* subfamily and *Jerseyvirus* genus. This taxonomic information was found to be consistent with the prediction from other tools like vConTACT2, ViPTree and VIRIDIC. Phages from the siphoviridae family including, PSDA-2, vB_SenS_SE1, ZCSE-6, S55, SHWT1, vB_SalP_LDW16, vB_SenS_TUMS_34 were well-recognized as promising biocontrol agents as described in literatures^31,45,48–51^. Despite the pan-genome analysis provides important insights into the genetic diversity at the species level, studies or pan-analysis on bacteriophages are rare. Pan-genome analysis of 65 *Salmonella* phages identified 4 core genes and 7 softcore genes that together comprised 2% of all genes. Our finding was in alignment with a study that reported the identification of 2.1% core genes during the pan-genome analysis of 100 phages from the Markadamsvirinae subfamily^24^. Overall, genomic analysis of our phage and subsequent comparisons with other *Salmonella* phages indicated the safety and suitability of L223 in biocontrol.

We examined the efficiency of phage L223 in reducing the counts of *Salmonella* Typhimurium in artificially contaminated chicken breast. Bacterial counts were significantly reduced in phage-treated meat at three different incubation times compared to phage-free control. During this experiment, we selected a MOI of 1 because bacteriolytic activity was consistent at that MOI as evident from the optical density in the time-kill curve assay. A 1.24, 2.17, and 1.55 log CFU/piece reduction in *Salmonella* Typhimurium was achieved when phage-treated meats were incubated for 2, 4, and 6 h, respectively at room temperature. Similar to our findings, Zhou et al., 2021 reported a significant decrease in *Salmonella* Enteritidis count after 6 h of treatment with *Salmonella* phage SapYZU01 at MOI = 1 at 25 °C^52^. A comparison of existing studies suggested that several factors including incubation time and temperature, a mixture of multiple phages, mode of phage applications, the multiplicity of infection, type of food matrices, and *Salmonella* serotype might influence the success of phage treatment in food products. For example, Sun et al., 2022 observed a positive correlation between the decrease in *Salmonella* Typhimurium and MOI, in which phage PSDA-2 reduced *Salmonella* contamination in chilled mutton within 120 h of treatment^31^. Reviera et al., 2022 isolated and characterized a *Salmonella* phage STGO-35-1 that decreased *Salmonella* Enteritidis counts by 2.5 log in chicken meat at 4 °C^39^. Another phage LPST94 also caused considerable reduction of *Salmonella* viable counts on chicken breasts with MOI = 1000 and MOI = 10,000 at 25 °C^53^. However, a phage cocktail is proven to be more effective in controlling pathogens compared to individual phages^54^. It also broadens phage host range as well as suppresses anti-phage resistance of pathogens. For instance, treatment of contaminated chicken breast with a cocktail of five phages at MOI = 10^4^ reduced the number of *Salmonella* Typhimurium by 2.0, 1.9, and 2.2 log CFU/piece at 2, 4, and 6 h, respectively^4,22^. Gvaladze et al., 2024 sprayed a cocktail of six phages on chicken skin contaminated with *Salmonella* Enteritidis and the mixed culture and achieved after 30 min a significant 1.8 log and 1 log reduction, respectively^55^. Spricigo et al., 2013 observed that dipping chicken breast for five minutes in a solution containing the bacteriophage cocktail and subsequently chilled at 4 °C for seven days showed significant 2.2 and 0.9 log10 cfu/g reductions in the concentrations of *Salmonella* Typhimurium and *Salmonella* Enteritidis, respectively^56^. In comparison, we assume that optimization of those influencing factors might enhance the bacteriolytic capability of L223 and make it more competent for *Salmonella* control in food and food products.

## Materials and methods

### Host strain and culture condition

*Salmonella* Typhimurium ATCC14028 was used as a host for bacteriophage isolation and characterization. Previously isolated *Salmonella* Typhimurium, *Salmonella* Enteritidis and other *Salmonella* spp. from poultry excreta and eggshells were utilized for phage host range determination^28^. All strains were grown on nutrient agar at 37 °C for 20–24 h. 20% glycerol stock (v/v) of all strains was kept at − 20 °C for storage.

### Isolation and propagation of bacteriophage

Five sewage samples were collected from different points of the drainage system of the Kaptan Bazar area, one of the largest retail poultry markets in Dhaka city. Then, samples were subjected to centrifugation at 10,000 rpm for 10 min at 4 °C to remove large particles and the resulting supernatants were filtered through a 0.22 µm syringe filter. Subsequently, 10 ml of filtrate was added to 30 ml log phage *Salmonella* Typhimurium ATCC14028 (host) culture and incubated overnight at 37 °C. The mixture was again centrifuged at 10,000 rpm for 10 min at 4 °C. Then, the double overlay agar method was used to isolate lytic phage. To do that, 100 µl filtered supernatant was mixed with 100 µl host and added to 0.7% semi-solid agar. Then, the mixture was poured onto prepared nutrient agar (1.5%) and incubated overnight at 37 °C. The bacteriophage’s capacity to produce plaque was observed and plaque-forming units (PFU) were counted. For phage purification, a sterile micro-pipette tip was first inserted into the center of the plaque and swirled to extract a single plaque. The plaque was then pipetted into 100 µl of normal saline and subjected to plaque assay. This procedure was repeated three consecutive times using the same host to obtain host-specific phage. In addition, the same assay was also followed using serially diluted purified bacteriophage to enumerate the number of phages (PFU/ml).

### Temperature and pH stability

The pure phage suspension was heat treated at different temperatures ranging from 20 to 80 °C for 1 h to assess the temperature stability of the phage. For determining the pH stability of phage, nutrient broths were prepared by adding NaOH or HCl to maintain pH from 2 to 12. After treatment, phage titers were measured by the double-layer method.

### Host range determination

Different *Salmonella* isolates along with other Gram-negative bacteria including *Escherichia coli*,* Vibrio cholerae*,* Pseudomonas aeruginosa*,* Acinetobacter baumannii*, and *Staphylococcus aureus* were tested for phage susceptibility. A 100 µl exponential phase culture of host bacteria was plated by double layer method and allowed the top layer to solidify. Then, a drop of 10 µl phage suspension (∼ 10^9^ PFU/ml) was spotted on the bacterial lawn and incubated at 37 °C for 24 h. The results of bacterial lysis were recorded to determine the host range. In addition, the efficiency of plating (EOP) was determined against these isolates using plaque assay as described by Hosny et. al.^57^. The average of PFUs on test isolates was divided by the average of PFUs on host bacteria to calculate EOP.

### Time-kill curve assay

Characterization of bacterial susceptibility to phage infection at different multiplicities of infection (MOI’s) was done by time-kill analysis^24^. Briefly, the optical density of the freshly grown culture of *Salmonella* Typhimurium ATCC14028 was set to OD_600_ = 0.6 (∼10^8^ CFU/ml). Then, bacterial culture was infected with different concentrations of phage to obtain final MOIs of 1.0, 0.1, 0.01, 0.001, and 0.0001 in a 96-well microplate and incubated at 37 °C. The optical density was measured at 20 min intervals for 360 min (6 h) using Promega GloMax EXPLORER.

### One step growth curve analysis

The latent period and burst size of phage L223 were determined through one step growth curve analysis as described by Kropinski et al*.* with some modifications^58^. Briefly, the host culture was mixed with phage suspension at MOI = 1. The mixture was incubated at 37 °C for 10 min to allow phage absorption. Then, the mixture was centrifuged at 10,000 rpm for 5 min. The resulting pellet was resuspended in 10 ml fresh nutrient broth and kept at 37 °C in an orbital shaker incubator. The phage titer was estimated at 10-min intervals for 60 min by standard plaque assay as described earlier. The time gap between absorption and the start of the first burst was termed the latent period. The burst size was calculated by dividing the final titer of the bacteriophage by the initial titer.

### Phage DNA extraction, sequencing and assembly

Bacteriophage DNA was extracted using a DNeasy Blood and Tissue kit (Qiagen, Germany) with some modifications as described by Jakociune et al.^59^. The concentration and purity of DNA were determined with a Nanodrop spectrophotometer and electrophoresis on 1% agarose gel. Then, the extracted phage DNA was subjected to paired-end sequencing using Illumina NextSeq2000 at the Bangladesh Council of Scientific and Industrial Research (BCSIR), Dhaka. The quality of raw reads was inspected with FastQC (https://www.bioinformatics.babraham.ac.uk/projects/fastqc/). The reads were trimmed using the fastp v0.23.4 (https://github.com/OpenGene/fastp)^60^ to remove adapters, sequence duplication and low-quality sequences. Finally, SPAdes v3.15.5 (https://github.com/ablab/spades)^61^ was used to assemble quality filtered reads with careful option. QUAST (https://github.com/ablab/quast)^62^ was employed to observe the characteristics of the assembled genome. In addition, the completeness of the phage genome was calculated using CheckV (https://bitbucket.org/berkeleylab/checkv/src/master/) v1.0.3^63^.

### Bioinformatic analysis of phage genome

The phage genome was annotated using the RAST (Rapid Annotation using Subsystem Technology) (https://rast.nmpdr.org)^64^ which was further verified using the blastp with an e-value = 0.01. Proksee (https://proksee.ca/)^65^ was used to create a circular genome map. The closely related phages were identified with blastn^66^ and ViPTree (https://www.genome.jp/viptree/) server (version 4.0)^67^. The phage lifestyle (lytic or temperate), the presence of tRNA were predicted by tRNAscan-SE (version 2.0) (http://lowelab.ucsc.edu/tRNAscan-SE/)^68^ and BACPHLIP (default version) (https://github.com/adamhockenberry/bacphlip)^69^, respectively. Moreover, ABRicate (https://github.com/tseemann/abricate) was used to scan phage genome for bacterial virulence factors and antimicrobial resistance genes using the VFDB^70^, CARD^71^ and ResFinder^72^ databases. VIRIDIC (https://rhea.icbm.uni-oldenburg.de/viridic/)^73^ calculates intergenomic similarities between viral genomes and therefore, was employed in the comparative genome analysis of the assembled phage with its close homologs. The figure of genomic comparison was generated using a freely available tool, Easyfig.

VICTOR (Virus Classification and Tree Building Online Resource) (https://ggdc.dsmz.de/victor.php#)^74^, a genome-based phylogeny and classification of prokaryotic viruses, was used for nucleotide-based grouping of phage. A circular proteomic tree was generated using VipTree that utilizes tblastx to compute genome-wide similarities and predicts taxonomic information of viruses and their hosts based on the Virus-Host DB. Moreover, vConTACT v2.0 (https://kbase.us/applist/apps/vConTACT/vcontact/release) was utilized for phage taxonomic assignment by performing shared proteome clustering analysis^75^. The association between the sequenced phage genome and other phage sequences on NCBI-GenBank was visualized using PhageClouds (https://phageclouds.ku.dk/)^76^, with a threshold of 0.2 for intergenomic distances. Finally, the pan-genome analysis of 65 *Salmonella* phages, belonging to the same family of *Salmonella* phage L223 with S_G_ ≥ 0.8 (VipTree) was performed using Roary v.3.11.2 (https://sanger-pathogens.github.io/Roary/)^77^.

### Application of bacteriophage

The isolated phage was applied on experimentally infected meat to assess its ability to reduce *Salmonella* Typhimurium ATCC14028 following the protocol described by Duc et al. with some minor modifications^22^. Commercially available chicken breast meat was purchased from the supermarket. The absence of *Salmonella* in meat samples was done by plating serially diluted homogenized meat on XLD agar. Then, the meat sample was cut into small pieces (3 × 3 cm). Both sides of the sliced meat were washed with 70% ethanol. Then, the meat pieces were placed onto a sterile petri plate and treated with UV for 1 h. 100 µl of bacterial suspension (10^8^ CFU/ml) was inoculated homogenously onto the meat samples. Then, the meat samples were kept at room temperature for 10 min in a biosafety cabinet to allow bacterial attachment. Bacteriophage titer was diluted to approximately 10^8^ PFU/ml to maintain the MOI = 1. 100 µl of bacteriophage suspension was applied on the surface of experimentally infected meat. For the meat pieces in the control group, 100 µl of normal saline was inoculated instead of bacteriophage. The meat pieces were incubated at 25 °C and bacterial count from both control and treatment groups was taken at 2, 4 and 6 h of incubation. The meat pieces were homogenized with 5 ml of saline. The serially diluted homogenate was spread on XLD agar and incubated at 37 °C for 24 h. Following incubation, viable counts of *Salmonella* in the control and treatment meat pieces were compared to determine phage treatment efficiency.

### Statistical analysis

All the experiments were conducted three times. Bacterial counts (log CFU/ml) and phage plaques (log PFU/ml) were tabulated as mean ± SD (standard deviation). The differences between treatment and control groups were determined by t-test using Prism 8 (GraphPad). *p*-value less than 0.05 was considered statistically significant.

## Conclusion

In this study, we isolated a *Salmonella* phage L223 that exhibited good physicochemical stability, a short latent time with a high burst size, and a strictly lytic lifestyle. The analysis of the phage genome indicated the safety of L223 in biocontrol applications. Finally, an assessment of the antibacterial efficacy in chicken meat resulted in significant reductions in *Salmonella* Typhimurium ATCC14028 counts. Despite these promising findings, the lack of data on the optimization of parameters during L223 application on meat remains a limitation of the study. Experimentation with an extended range of MOI, time and temperature might provide more insights into the suitability of the therapeutic supplementation of L223. However, taken together, the findings of our study will initiate the development of phage-based products to combat MDR *Salmonella* in the food industry in Bangladesh.

## Data Availability

The complete genome sequence of Salmonella phage L223 is available on NCBI Genbank under the accession number PP034127.
